# Pseudo-Perception: A New Concept for Resolving the Problems of Long Instruments in Neurosurgical Interventions

**DOI:** 10.7759/cureus.73697

**Published:** 2024-11-14

**Authors:** Moneer K Faraj, Siham Sabah Abdullah, Hussein Hade Saeed, Ali Al-Zuky, Mustafa Ismail

**Affiliations:** 1 Department of Surgery, College of Medicine, University of Baghdad, Baghdad, IRQ; 2 Department of Physiology and Medical Physics, College of Medicine, Al-Nahrain University, Baghdad, IRQ; 3 Department of Physics, College of Science, Mustansiriyah University, Baghdad, IRQ; 4 Department of Surgery, Baghdad Teaching Hospital, Medical City Complex, Baghdad, IRQ

**Keywords:** brain endoscope, long instruments, pseudo-perception, surgical instruments, tremors

## Abstract

Long surgical instruments, particularly in brain endoscopy, often compromise precision and control due to the physical distance between the surgeon's hand and the instrument’s tip, increasing the likelihood of tremors. Various technological solutions, including robotics, have been proposed to address this issue. This report outlines the development of a pseudo-perception system aimed at improving control over long instruments in neurosurgical procedures by manipulating visual feedback to enhance the surgeon's sense of proximity to the instrument’s tip.

The pseudo-perception system uses real-time image manipulation via MATLAB software to fuse images of the surgeon's hand and the instrument's tip. This is achieved by recording the surgeon's hand movements and superimposing them onto the endoscopic view. The system is designed to visually trick the surgeon’s brain into perceiving the hand as being closer to the surgical field, thereby improving precision without requiring expensive robotic systems. The system was developed and tested in a simulated environment with standard neurosurgical instruments. Initial observations suggest that the pseudo-perception method improves manual precision and reduces tremors. However, this system is still in the prototype stage and requires further technical refinement and testing in real-world settings.

This report provides a technical overview of the pseudo-perception system, offering a potential low-cost solution for improving control with long instruments in neurosurgery. Future developments will focus on enhancing the system’s accuracy and usability in clinical practice.

## Introduction

It looks quite good whenever we try to write with a conventional pen. Still, if we use a long stick, the result will be awkward. Using long tools is sometimes mandatory, but the hand's precision will be reduced since the target point, i.e., the tip of the working instrument, is far from the controlling surgeon's hand. Also, the long tools will provoke tremors in the hand. Many trials have been conducted to improve the use of these long instruments, e.g., the use of robots, in which the robots perform the work while the surgeon is away from the surgical field [1-4].

In neurosurgery, the use of long instruments, particularly in brain endoscopic procedures, poses significant challenges in terms of precision and control. Long tools, by their nature, extend the physical distance between the surgeon's hand and the target tissue, which increases the likelihood of tremors and reduces fine motor control. This phenomenon can be compared to the awkwardness one experiences when trying to write with a long stick instead of a pen. The further the tip of the instrument is from the surgeon's hand, the greater the reduction in manual precision, which can hinder the overall efficacy of the surgery [5]. Various approaches have been explored to mitigate these issues. Robotic systems, for instance, offer an innovative solution by filtering hand tremors and enabling enhanced precision in handling surgical instruments. Robotic control systems, combined with advanced imaging modalities such as intraoperative 3D ultrasound (iUS) and intraoperative magnetic resonance imaging (iMRI), have greatly improved precision in neurosurgical procedures. These technologies provide real-time feedback, allowing the surgeon to adjust movements with unparalleled accuracy, thereby addressing the inherent limitations of human dexterity [6].

Our work introduces the concept of "pseudo-perception," a novel approach designed to enhance the surgeon's sense of proximity to the instrument's working tip, thereby improving precision and reducing tremors without the need for advanced robotics. By utilizing a real-time visual fusion of the surgeon’s hand and the instrument tip via computational methods, we aim to deceive the brain into perceiving the instrument as an extension of the hand, effectively bridging the gap introduced by long tools. This method addresses the manual precision issues experienced with conventional instruments without requiring the complex infrastructure of robotic systems.

## Technical report

Technical approach

**Figure 1:**
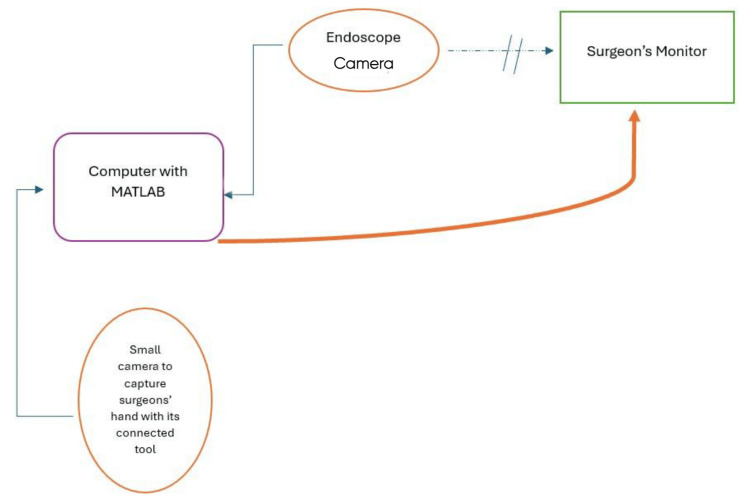
The main scheme of the pseudo-perception system involves dislodging the camera cable from the surgeon's monitor and connecting it to the computer, which receives another cable from a camera mounted to monitor the surgeon's hand. With the processing of the two images using MATLAB software, we properly fused them and sent them to the surgeon's monitor.

The setup modifies the surgeon's visual feedback, making it appear as though their hand is closer to the tip of the surgical instrument, thereby improving control and minimizing the effects of hand tremors, particularly when using long instruments (Figure 1).

Equipment setup

For this study, we utilized a standard Storz® training station designed for brain endoscopic procedures. The station was equipped with a brain endoscope camera, a display monitor, and long biopsy instruments measuring approximately 45 cm in length. These long tools are frequently necessary for neurosurgical interventions but often reduce the surgeon’s manual precision due to the physical distance between the hand and the working tip of the instrument. To address this issue, our experimental setup altered the traditional method of connecting the endoscope to the display, allowing us to manipulate the visual feedback provided to the surgeon.

Endoscopic video feed modification

In the traditional setup, the camera of the brain endoscope is connected directly to the screen display. In our experiment, the camera's cable was passed through the computer, where the real-time video was processed by MATLAB before being output to the monitor. This modification allowed us to process and manipulate the video feed, which is also the core basis for our pseudo-perception technique. The image from the endoscope was projected onto a computer screen, where it was manipulated, then projected directly onto the monitor in front of the surgeon.

Hand image integration

Other than the endoscopic view, another camera was incorporated to capture the surgeon’s hand performing the operation. The video provided by this camera is processed in MATLAB, where only those frames that contain the surgeon’s hand are extracted. The images of the hands are superimposed on the endoscopic view of the surgical field. Transparency for the hand was set so that it would not interfere with the surgical field, allowing a clear view of the operative site to be obtained. In this way, it created the illusion of the surgeon’s hand being near the working tip of the instrument - a requirement necessary to enhance precision in fine neurosurgical operations.

Alignment and synchronization

**Figure 2:**
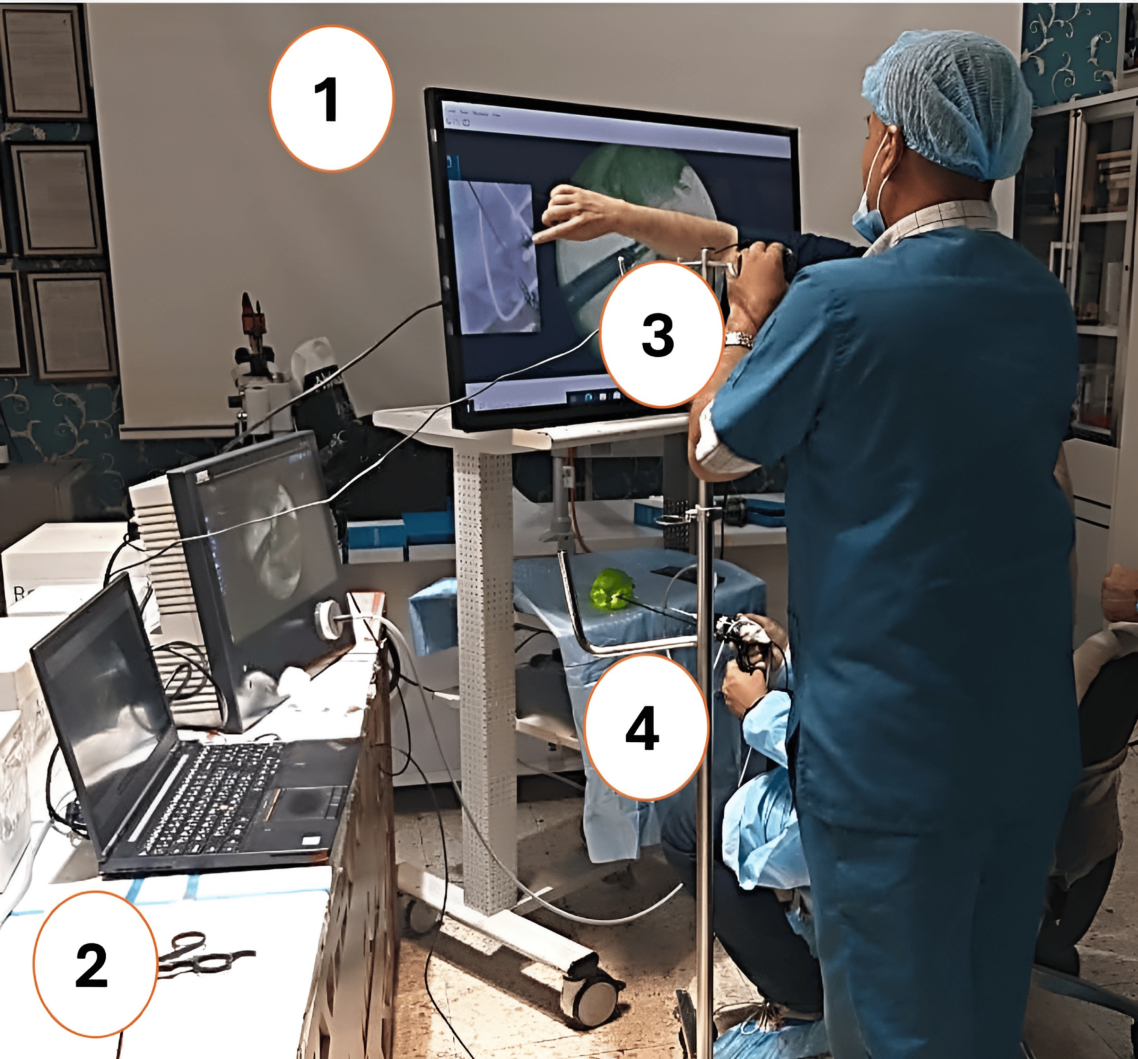
The system is composed of the surgeon's monitor (1), the computer that receives cable of the camera first with MATLAB software facility (2), another camera held by an assistant to record real-time the movement of the surgeon's hand (3), the long instruments used by the surgeon to pick up the seeds from the green pepper (4).

In these experiments, to maintain visual coherence between the surgeon's hand and the surgical instrument, the image of the hand was rotated and aligned with the orientation of the tip of the instrument in the endoscopic view. The moved image of the hand would then move in sync with the tip of the instrument as the surgeon moved his hand, creating an almost real-time visual feedback loop (Figure 2).

**Figure 3:**
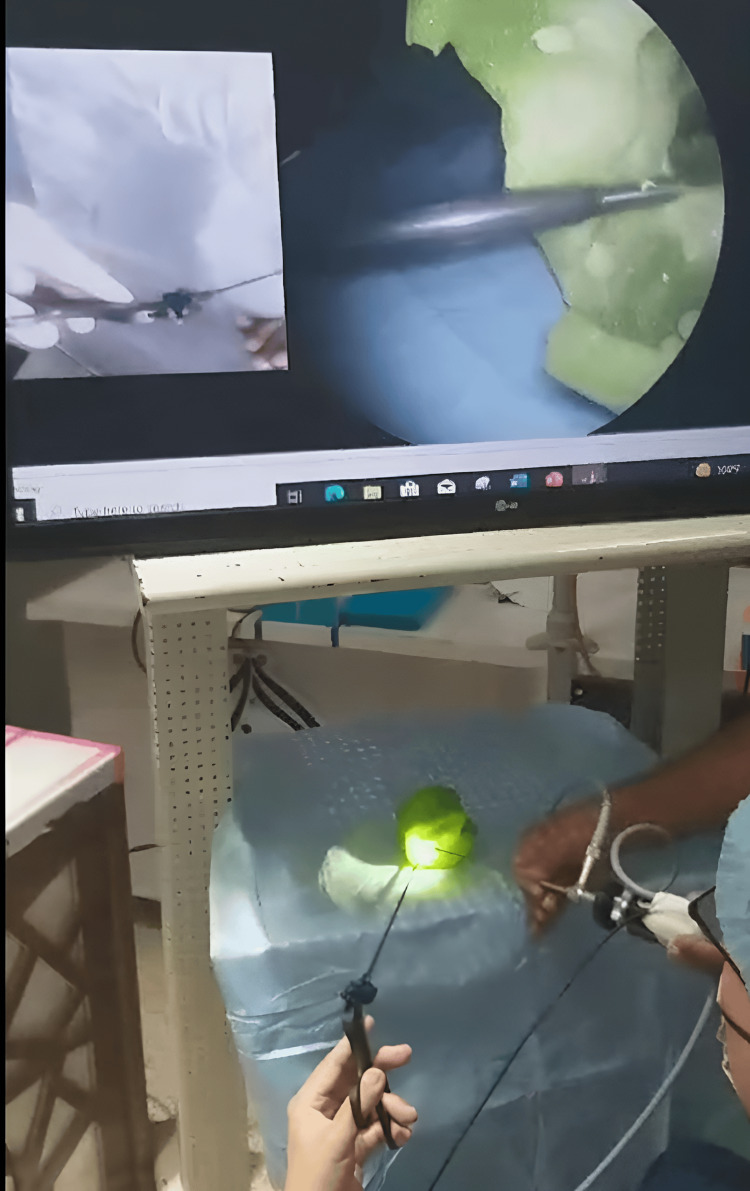
The fused photos with the hand of the surgeon in line with the tip end of the biopsy forceps.

This alignment tricked the surgeon's brain into thinking their hand was closer to the working tip than it really was, thereby increasing their control over the instrument and diminishing the effects of hand tremor (Figure 3).

System performance and observations

During the development of the pseudo-perception system, several performance aspects were closely observed in a controlled, simulated environment. The system was designed to assist surgeons using long instruments in neurosurgical procedures by creating a visual illusion that brings the hand closer to the working tip of the instrument.

Precision and accuracy

One of the key objectives of the pseudo-perception system was to enhance the surgeon's ability to perform precise movements, particularly during intricate neurosurgical tasks. The visual trick provided by the system, which fused the surgeon’s hand movements with the endoscopic view of the surgical field, allowed participants to feel as though their hands were physically closer to the target tissue.

Reduction in tremors and improved control

One of the most significant challenges when using long instruments is the magnification of hand tremors due to the physical distance between the surgeon's hand and the working tip of the instrument.

The system’s ability to simulate closer proximity between the hand and the instrument helped mitigate the tremor effect, thereby offering better control. This was especially valuable during tasks requiring continuous stability, such as gripping or dissecting delicate tissue. By minimizing the perception of distance, the system effectively allowed surgeons to stabilize their hand movements, resulting in smoother and more controlled actions.

Task efficiency

Efficiency in surgical procedures is often measured by the time taken to complete a given task without compromising precision. In the simulated environment, the pseudo-perception system demonstrated the potential to reduce task completion time. Surgeons were able to perform simulated biopsies and similar tasks faster than they could with conventional setups.

While formal time comparisons were not part of the primary objective, it was generally observed, that the intuitive design of the pseudo-perception system allowed participants to adapt quickly.

## Discussion

The application of pseudo-perception technology demonstrated that the time required to perform tasks with long instruments was drastically reduced, thereby greatly improving surgeons' efficiency at a fraction of the cost compared to more complex systems [7]. Although this is promising, this method is still somewhat in its infancy and requires further development. Advanced features, including high-resolution cameras with motion detection (so that hand movement is detected quite accurately), and more sophisticated software supporting real-time image subtraction, would make this a far more capable system. Needless to say, all these developments require resources and skills presently beyond those available in our laboratory. It needs continuous innovation and support in this respect.

Sophisticated microsurgical robotics has greatly improved surgical precision and minimized the challenges imposed by hand tremors in such fine procedures. Systems like the Micron handheld instrument have been designed to actively cancel out tremors, providing real-time compensation for involuntary movements, hence increasing accuracy in such microsurgical tasks as retinal vein cannulation. These developments constitute a huge leap forward in reducing the margin of human error in surgery, especially in manipulating long instruments, which are particularly prone to amplifying tremors [8]. Besides, some robotic-assisted systems have integrated various feedback technologies, including filtering of tremor and scaling of motion, enabling more finessed control over procedures in which even the slightest tremor could constitute major damage [9]. Comparing these to our pseudo-perception technique, it is observed that, while robotics does present an accuracy no other modality can match, the method here presents a cost-effective alternative for neurosurgery tremor reduction and increased control sans complicated machinery.

Our study suggests the possibility of the ability of pseudo-perception technology to improve the precision and control necessary in neurosurgery with long instruments. The modification of visual feedback by the system reduces hand tremor, thus enhancing fine manual dexterity, as manifested by the faster completion times of tasks and fewer faulty trials during the trials.

The pseudo-perception system significantly reduced both the completion time and the error rate of tasks, as compared to the conventional approach. This demonstrates the ability of the system to close the gap between the hand of the surgeon and the instrument tip for finer control during critical maneuvers. The enhanced feel of proximity thus allowed surgeons to overcome the inherent tremor with long instruments, leading to higher precision with fewer errors. This is an important aspect of neurosurgery, whereby even small inaccuracies may lead to grave consequences in patient outcomes.

The pseudo-perception system is, however, quite powerful; further development may not go amiss. In a system with added, more sophisticated devices, such as cameras able to detect and track hand movements in motion, more detail could be added to the real-time feedback coming from the system. Of course, all these enhancements require resources and capabilities beyond our laboratory's means. It would, therefore, be very worthwhile to continue collaboration and create future iterations of this system with more technologically advanced laboratories.

With these limitations, the pseudo-perception system is an affordable and easy alternative to utilizing a robotic system in neurosurgery. While the latter promises far better precision, it is very costly; furthermore, its complicated setup may not be possible in all surgical settings. In contrast, pseudo-perception requires very minimal additional equipment and, hence, is a good option to help improve surgical precision in resource-limited setups. In addition, pseudo-perception provides an intuitive interface where surgeons do not need to drastically change their traditional techniques. Therefore, surgeons can adapt quickly and apply it clinically within a much shorter period of time.

The pseudo-perception technique provides a novel solution to long-instrument challenges within the complex work of neurosurgery and extends control and precision beyond this by reducing hand tremors via an easily accessible and low-cost approach. In continuing the refinement of this technology in the field, we foresee wider clinical applications to improve surgical outcomes across a range of settings. This will involve future research being directed toward developing the technological potential of the system, so that it may maximize its capabilities for improving surgical accuracy.

## Conclusions

Pseudo-perception technology offers a promising solution to the challenges of using long instruments in neurosurgery by improving precision and reducing hand tremors. This method significantly enhances task efficiency and control, providing a practical and cost-effective alternative to complex robotic systems. While further development is needed - such as integrating motion-detecting cameras and more advanced software - pseudo-perception has the potential to become a valuable tool in both resource-limited and high-tech surgical environments. As we continue to refine this technology, we anticipate its broader application in neurosurgical procedures, particularly in scenarios where precision and control are critical. Future research will focus on addressing the limitations identified in this study, with the goal of optimizing the system for clinical use and improving patient outcomes in neurosurgical interventions.
